# Cognitive behavioural therapy for menopausal symptoms: a systematic review of efficacy in improving quality of life

**DOI:** 10.1186/s12905-025-04142-y

**Published:** 2025-12-29

**Authors:** Hopewell Rukure, Margaret Husted

**Affiliations:** 1 Hampshire and Isle of Wight Healthcare NHS Foundation, Winchester, UK; 2 Engaging Communities Research Centre, University of Winchester, Winchester, UK

**Keywords:** Menopause, Cognitive behavioural therapy, Women’s health, Quality of life

## Abstract

Menopause, whilst a natural biological transition, can cause a range of symptoms that have a significant, negative impact on some women’s quality of life. Psychological interventions have been proposed as a means of alleviating the detrimental impact of menopausal symptoms and supporting women’s coping capacity during this period. This systematic review evaluates the efficacy of Cognitive Behavioural Therapy (CBT) in improving menopausal symptoms such as hot flushes, night sweats, anxiety, depression and sleep disturbances and enhancing quality of life. The review covers research published from 1990-Dec 2024 and included randomised control trials, quasi-experimental and observational study designs. A total of 16 studies involving 910 women were identified and analysed, encompassing various CBT formats including group, individual, and self-help interventions. Results demonstrate that CBT significantly improves health-related quality of life and alleviates vasomotor, psychological, and sleep-related symptoms. Group-based CBT yielded the most substantial benefits, while self-help modalities showed moderate but meaningful improvements. The findings underscore CBT’s role as a viable non-pharmacological intervention for menopausal symptom management. This is particularly valuable where hormone replacement therapy is contraindicated or declined. As access to in-person psychological interventions are not available to all, future research should explore whether digital CBT platforms can be effectively adopted to enhance accessibility and scalability.

## Introduction

Menopause, typically occurring between the ages of 45 and 55, is a natural biological transition marked by a decline in reproductive hormones, particularly oestrogen [21]. While natural, menopause can lead to a spectrum of symptoms, including vasomotor disturbances (e.g., hot flushes and night sweats), sleep disturbances, mood swings, anxiety, and depression [21]. In addition to vasomotor and psychological symptoms, menopause can also involve sexual dysfunction (e.g,. vaginal dryness, reduced libido), cognitive changes and physical discomfort such as joint pain and fatigue [21]. Each of these symptoms can profoundly affect a woman’s quality of life [54]. Menopausal women often report lower health-related quality of life scores, higher absenteeism from work, and reduced productivity compared to their premenopausal peers [12]. Studies estimate that menopause-related symptoms can lead to productivity losses of billions of dollars annually [12]. Given the widespread prevalence of these symptoms and their significant impact, there is a need to explore effective interventions that address both the physiological and psychological challenges posed by menopause.

Hormone replacement therapy (HRT) has been a cornerstone of treatment for vasomotor symptoms like hot flushes and night sweats, by replenishing levels of oestrogen and if required can also be combined with progesterone. The benefits of HRT include improved mood, prevention of osteoporosis, and significant relief from hot flushes [41], with indications of insufficient evidence to recommend HRT for dementia prevention [36]. However, concerns about its long-term safety, particularly regarding its association with breast cancer [6] and cardiovascular risks [62], persist. Evidence indicates that although some risks are present, the picture is complex with indications that HRT may be protective of some cancers [60] and cardiovascular risk may only relate to venous thromboembolism and vary depending on HRT administration route [18].

Given the complex picture surrounding the risks associated with HRT, there is interest in non-pharmacological interventions [52], particularly psychological therapies like cognitive behavioural therapy (CBT) as a means for providing support. CBT is a structured psychotherapy focused on modifying maladaptive thinking patterns and behaviours to improve functioning. It has been widely studied and practised for conditions such as depression, anxiety, and insomnia, and more recently, its application has expanded to managing menopausal symptoms [8], where it has emerged as a promising intervention for addressing the psychological and somatic symptoms of menopause [26]. CBT tailored explicitly for menopause aims to help women develop coping strategies to manage vasomotor symptoms, mood disturbances, and sleep issues. The therapy addresses cognitive distortions related to menopause (e.g., the belief that symptoms are uncontrollable or shameful) and employs techniques such as relaxation training, cognitive restructuring, and paced breathing [44].

Research has shown that CBT can significantly improve wellbeing for menopausal women. For example, Mollaahmadi et al. [44] demonstrated that face-to-face CBT, particularly group-based therapy, was effective in reducing vasomotor symptoms like hot flushes and night sweats, as well as improving psychological outcomes such as anxiety, depression, and sleep quality. Additionally, CBT has also been associated with improved overall mental health and functioning, offering a holistic approach to managing the multifaceted symptoms of menopause [8].

While CBT is beneficial, other approaches such as mindfulness-based therapies, yoga, and lifestyle modifications have also been explored for menopause management. Mindfulness-Based Cognitive Therapy (MBCT), which combines CBT with mindfulness practices, has been found to reduce the severity of menopausal symptoms (including hot flushes and night sweats) by promoting better emotional regulation and stress management [37]. Yoga, as a body-based mindfulness practice, may support physical and mental well-being by improving body awareness and reducing stress [29]. Additionally, lifestyle modifications such as regular physical activity, a balanced diet, and adequate sleep contribute to overall health and may alleviate some menopausal symptoms [15]. However, despite the growing interest in these non-pharmacological approaches, the evidence supporting the effectiveness remains mixed. For instance, while MBCT has demonstrated benefits in reducing menopausal symptoms, some studies suggest that its effects may be modest and more pronounced in women undergoing natural rather than treatment-induced menopause [37]. Similarly, although yoga is often associated with improved well-being, systematic reviews reported inconsistent outcomes, with some high-quality trials showing no significant improvement on vasomotor symptoms [29]. Lifestyle modifications are widely recommended for general health,however, the direct influence on menopausal symptoms such as hot flushes and night sweats is not consistently supported by clinical evidence [15]. These findings highlight the importance of an evidence-informed approach when integrating complementary therapies into menopause care.

Recent clinical guidelines support the role of CBT in menopause care. The British Menopause Society [7] recommends CBT and clinical hypnosis as effective non-hormonal treatments for managing vasomotor symptoms. The North American Menopause Society [48] supports CBT as a first-line non-hormonal option for vasomotor and psychological symptoms. In 2024, the National Institute for Health and Care Excellence (NICE) updated its guidance to recommend menopause specific CBT for hot flushes and night sweats, alongside HRT or as an alternative for those who cannot or choose not to use HRT. NICE [47] recommends CBT for sleep problems and depressive symptoms associated with menopause.

Several systematic reviews have previously examined CBT for menopausal symptoms. Ye et al., [63] conducted a meta-analysis and found moderate to large effects of CBT on vasomotor and psychological symptoms across diverse populations. Chang et al. [11] focused on breast cancer survivors and reported CBT’s efficacy in alleviating treatment-induced menopausal symptoms. Spector et al. [58] systematic review, concluded that CBT is effective in reducing vasomotor symptoms and improving HRQol when delivered in group formats. These findings provide a strong evidence base for CBT as viable intervention. These reviews collectively reinforce the growing consensus that CBT is a viable non-hormonal intervention. However, the variability in efficacy by intervention type underscores the need for research to evaluate the efficacy of various non-pharmacological interventions to determine which interventions should or should not be recommended as standard. Given the interest and support for CBT as an effective intervention for menopausal symptoms, it is crucial to systematically assess the breadth of evidence and determine CBT’s overall efficacy in improving quality of life for menopausal women. This review aims to consolidate findings from randomised controlled trials (RCTs), quasi-experimental studies, and observational studies to comprehensively evaluate the impact of CBT on menopausal symptoms, focusing on quality of life, psychological well-being, and vasomotor symptom management. By addressing this gap in the literature, the review can inform clinical practice and support women navigating this impactful life transition.

## Methods

### Study design

This systematic review was conducted in accordance with the PRISMA (Preferred Reporting Items for Systematic Reviews and Meta-Analyses) guidelines to ensure transparency and reproducibility [43]. The review considered evidence from randomised controlled trials (RCTs), quasi-experimental studies, and observational studies investigating the efficacy of CBT interventions in improving the quality of life, psychological wellbeing (e.g., anxiety and depression), vasomotor symptoms, (e.g., hot flushes and night sweats) and sleep disturbances among menopausal women. This systematic review protocol was pre-registered with PROSPERO (Registration No. CRD42024510673). PROSPERO is an international database of prospectively registered systematic reviews in health and social care, which aims to provide transparency in the review process and help avoid unplanned duplication.

### Search strategy

A literature search was performed across multiple databases, including PubMed, Cochrane Library, Scopus, PsycINFO, and Google Scholar. The search covered publications from January 1990 through December 2024. Search terms included various combinations of “cognitive behavioural therapy and Mindfulness-based cognitive therapy” (and its abbreviation CBT, MBCT), provided they adhered to core CBT principles, “menopause,” “menopausal symptoms,” “quality of life,” and “vasomotor symptoms, 'hot flushes', 'night sweats', 'sleep problems', 'anxiety', and 'depression'.” Language restrictions were applied with a focus on studies conducted in English. Reference lists of relevant articles were screened to identify additional studies.

### Eligibility criteria

Inclusion and exclusion criteria were defined using the PICOS framework:

- Population: Women aged 40–65 years experiencing perimenopause or post-menopause.
- Intervention: CBT-based interventions (including standard CBT and mindfulness-based CBT programs).
- Comparison: Non-CBT interventions (e.g., lifestyle changes, relaxation techniques) or no treatment.
- Outcomes: Primary – improvement in quality of life measured with validated instruments (e.g., MENQOL questionnaire, SF-36 survey); Secondary – reductions in specific menopausal symptoms (vasomotor symptoms, anxiety, depression) and improvements in sleep disturbances.
- Study Design: RCTs, quasi-experimental studies, or longitudinal observational studies meeting the above criteria.

**Fig. 1:**
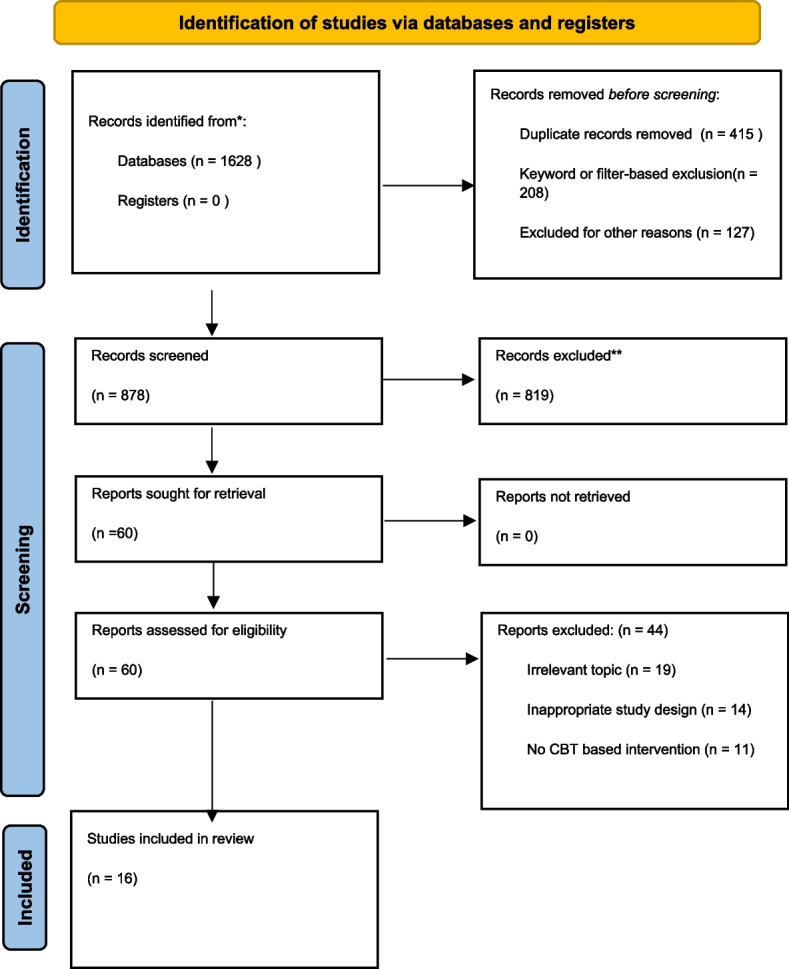
Prisma flow diagram illustrating the study selection process. Note: The diagram includes the number of records, identified, screened, excluded, and the final studies included, along with reasons for exclusions at each stage. Explanation of exclusion reasons. Irrelevant Topic: Studies that did not focus on menopausal symptoms or CBT interventions. Inappropriate study design: Studies that did not meet the methodological quality criteria set by the review. No CBT-based intervention: Studies that did not use CBT or combined treatment approaches as interventions

The PRISMA flow diagram (Fig. 1) below illustrates the study selection process, including the number of records identified, screened, excluded, and the final studies included, along with reasons for exclusion at each stage.

### Study selection

Two reviewers (HR and MH) independently screened the titles and abstracts of all retrieved records for relevance. Studies that appeared potentially eligible were retrieved in full text for detailed assessment. Any discrepancies between the reviewers were resolved through discussion. This selection process adhered to PRISMA guidelines to ensure a systematic and unbiased inclusion of studies. The initial database search yielded a total of 1,628 articles. After removing duplicates, ineligible studies etc., 878 studies were screened based on titles and abstracts. Following screening, 60 studies were identified as potentially relevant, and full texts were retrieved for detailed assessment. Sixteen studies met the inclusion criteria and were included in the systematic review. The primary reasons for excluding the other 44 full-text articles were an irrelevant focus on unrelated topics (*n* = 19), insufficient methodological quality (*n* = 14), or ineligible interventions not involving CBT (*n* = 11).

### Data extraction

Key data were extracted from each study using a standardised extraction form. The following information was collected from each included article:

- Study characteristics: authors, publication year, country, and study design.
- Population characteristics: sample size, age range of participants, and menopausal status (peri-, menopausal, or postmenopausal).
- Intervention details: type of CBT (individual, group, self-help, etc.), intervention duration, frequency of sessions, and delivery format (face-to-face, online, etc.).
- Comparison condition: details of the control or comparison group (e.g., treatment-as-usual, another intervention, or no intervention).
- Outcomes measured: quality of life metrics used and results, specific symptom outcomes (frequency/severity of vasomotor symptoms, mood/anxiety scores, sleep quality), and follow-up duration if applicable.

### Risk of bias and quality assessment

The risk of bias for each included RCT was assessed using the Cochrane Risk of Bias tool [24], evaluating the following domains:

- Random sequence generation (adequacy of randomisation method).
- Allocation concealment (whether group assignments were hidden from investigators).
- Blinding of participants and personnel (whether participants and researchers were blinded to treatment).
- Incomplete outcome data (whether there were missing data and how they were handled).
- Selective outcome reporting (whether all prespecified outcomes were reported).

For non-randomised studies, the GRADE (Grading of Recommendations Assessment, Development and Evaluation) approach was applied to rate the overall quality of evidence for key outcomes across studies [22].

### Data synthesis

A meta-analysis was conducted for studies that provided sufficient data to calculate pooled effect sizes using a random-effects model [24]. Standardized mean differences (SMDs) were used for continuous outcomes (e.g., quality of life scores), while odds ratios (ORs) were calculated for binary outcomes (e.g., symptom improvement) [12]. Heterogeneity between studies was assessed using the I^2^ statistic. Sensitivity analyses were performed to explore the robustness of the results by excluding studies at elevated risk of bias [44].

A narrative synthesis was conducted when a meta-analysis was not possible due to insufficient or heterogeneous data. Preplanned subgroup analyses were undertaken to compare the effects of different CBT formats (e.g., individual vs. group, face-to-face vs. online delivery) and to explore whether outcomes differed among specific subpopulations (such as natural menopause versus surgical menopause) [8].

### Outcome measures

#### Primary outcome

The primary outcome of interest was health-related quality of life in menopausal women, as measured by validated instruments such as the Menopause-Specific Quality of Life (MENQOL) questionnaire or the 36-Item Short Form Health Survey (SF-36). These tools capture multiple dimensions of quality of life (physical, psychological, and social well-being) by providing a comprehensive assessment of well-being.

#### Secondary outcomes

Secondary outcomes included the frequency and severity of specific menopausal symptoms and psychological health indicators. We examined changes in vasomotor symptoms (e.g., frequency of hot flushes and night sweats), mood and anxiety levels, and sleep quality. By evaluating these secondary outcomes, the review aimed to determine whether CBT not only improves general quality of life but also alleviates the key symptoms that contribute to diminished quality of life during menopause.

### Characteristics of included studies

The sixteen studies in this review comprised 910 menopausal women participants, with sample sizes ranging from 19 to 140. Studies were conducted across various geographical regions, including the United Kingdom (*n* = 5), the United States (*n* = 4), Iran (*n* = 3), Spain (*n* = 2), Canada (*n* = 1) and Switzerland (*n* = 1). The age range of participants was 45—65 years, covering women in perimenopausal, menopausal, and postmenopausal stages [8]. Of the included studies, ten were randomised controlled trials (RCTs), three were quasi-experimental, two were pilot studies, and one was a longitudinal observational study. Most studies evaluated the effects of CBT delivered in group formats (*n* = 9), but four studies used individual CBT, and three utilised self-help CBT with minimal therapist interaction. Table 1 below summarises the characteristics of included studies, including intervention format, control condition, primary outcome, and effect size metric. Of the 16 included studies, 13 studies used standard CBT and 3 used mindfulness-based CBT (MBCT).

**Table 1:**
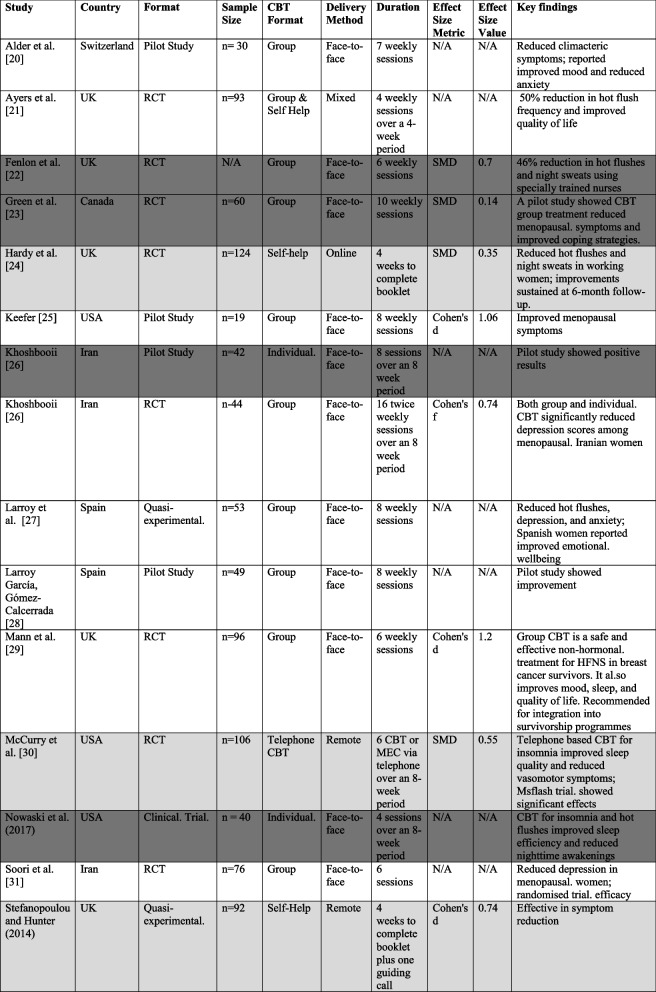
Characteristics of CBT interventions [1, 5, 17, 19, 23, 30, 31, 34, 35, 39, 42, 55]

Colour Shading: Light grey shading = Self Help; Dark grey shading = Individual; Non shading = Group and mixture of self help

*Abbreviations: CBT *Cognitive Behavioural Therapy, *MBCT *Mindfulness Based Cognitive Therapy, *SMD *Standardised Mean Difference, *MENQOL *Menopause specific Quality of Life Questionnaire

## Results

### Intervention characteristics

All interventions examined were based on CBT, though their format and delivery varied. Face-to-face group CBT was the most common format with session duration ranging from 60–120 min typically delivered once per week over 4–12 weeks. Individual CBT interventions followed similar session lengths and frequencies. Self-help CBT was provided via guided booklets or online modules with minimal therapist contact, with participants required to work through therapy exercises independently. Every intervention targeted both psychological symptoms (such as mood disturbances and anxiety) and vasomotor symptoms. Additionally, a subset of four studies explicitly addressed sleep problems and cognitive difficulties as part of the CBT program. Table 1 shows the Study Characteristics for included studies.

### Quality assessment and sensitivity analyses

**Fig. 2:**
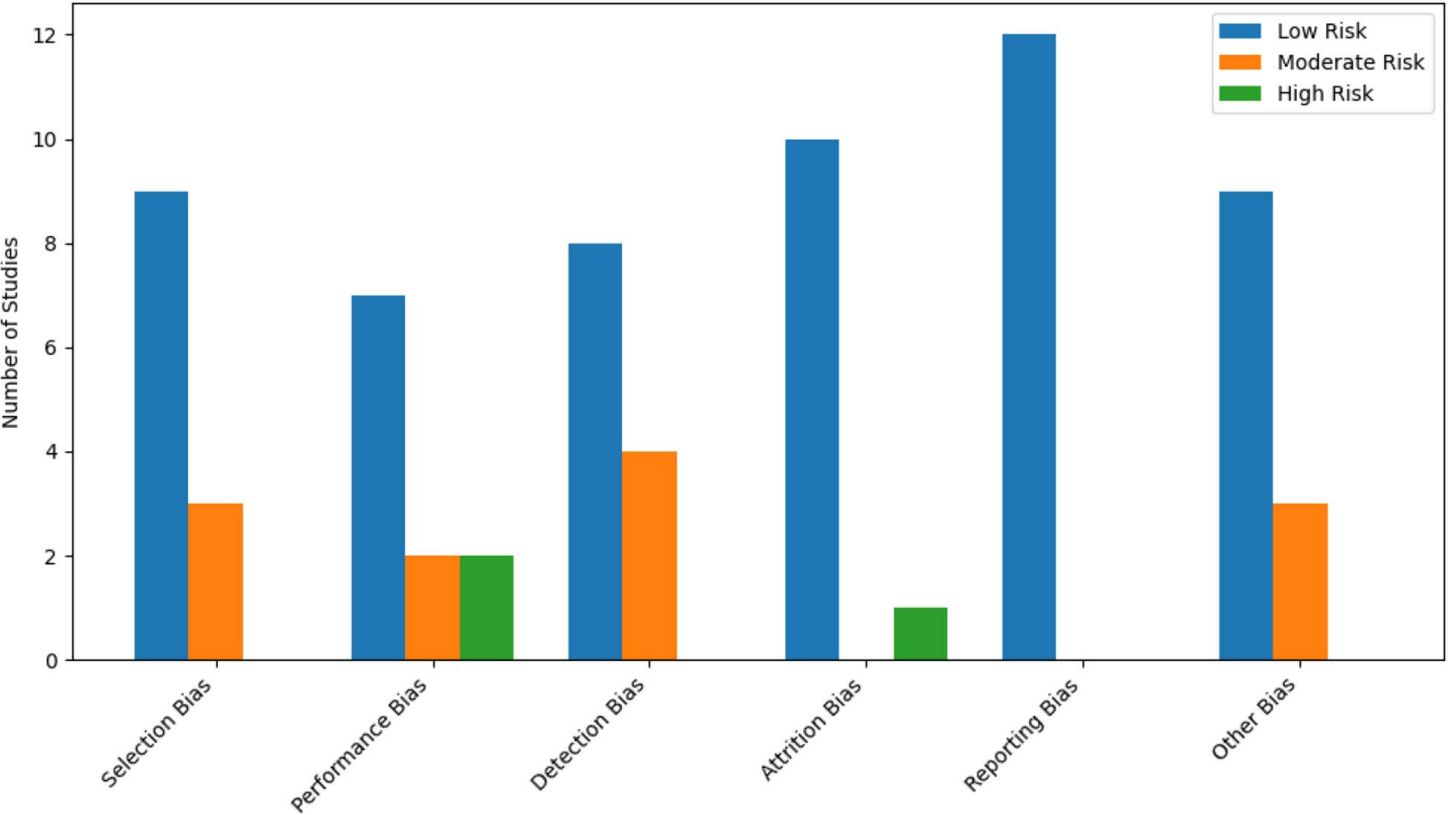
Cochrane risk of bias assessment across included studies. Note: The diagram summarises the risk of bias ratings across all included studies using the Cochrane framework

The risk-of-bias assessment indicated that studies had variable quality; nine studies were rated as high quality, seven as moderate quality, and none as low quality (see Fig. 2). The common potential biases in the studies.

- Performance bias, particularly in pilot and quasi-experimental studies to lack of binding of participants and personnel.
- Detection bias, where reliance on self-reported outcomes introduced moderate risk in several studies.
- Attrition bias, which generally low across studies, though a few pilot studies had higher dropout rates.
- Selection bias was mostly low with some concerns in non-randomised or pilot designs.

Three studies had high risk in at least one domain, primarily due to small sample sizes, lack of binding or high attrition. Observational studies such as Rodrigo et al. [12] were marked as “Not Applicable” for certain domains like performance and attrition bias. A sensitivity analysis excluding studies with moderate or high risk in multiple domains showed that overall effect sizes remained consistent. This supports the reviews conclusions and suggests that the positive effects of CBT are not driven solely by the highest-quality studies ( As shown in Tables 2 and 3).

**Table 2:** Combined CBT and HRT treatment

Study	Country	Sample Size	CBT Format	Delivery Method	Duration	Effect Size Metric	Effect Size Value
Manson et al.., 2024 [40]	USA	N/A	Combined CBT + HRT	Mixed	12 months	MENQOL Improvement	↑25% vs CBT al.one
Rodrigo et al.., 2023 [12]	UK	N/A	Group	Face-to-face	6 months	Percentage Reduction	Hot flushes ↓40%, Anxiety ↓50%, Depression ↓55%

*Abbreviations*: *CBT* Cognitive Behavioural Therapy, *HRT* Hormone Replacement Therapy, *MENQOL* Menopause specific Quality of Life Questionnaire

**Table 3:** Cochrane risk of bias assessment across included studies

Study	Format	Selection Bias	Performance Bias	Detection Bias	Attrition Bias	Reporting Bias	Other Bias	Overall Quality
Alder	Clinical Trial	Some Concerns	Some Concerns	Some Concerns	Some Concerns	Some Concerns	Some Concerns	Moderate Quality
Ayers et al	RCT	Low Risk	Low Risk	Low Risk	Low Risk	Low Risk	Low Risk	High Quality
Fenlon et al.	RCT	Low Risk	Moderate Risk	Low to Moderate Risks	Low Risk	Low Risk	Low Risk	High Quality
Green et al	Pilot Study	Some Concerns	Some Concerns	Some Concerns	Some Concerns	Some Concerns	Some Concerns	Moderate Quality
Hardy et al	RCT	Low Risk	Low Risk	Low Risk	Low Risk	Low Risk	Low Risk	High Quality
Keefer	Pilot Study	Moderate Risk	High Risk	Moderate Risk	Low Risk	Low Risk	Moderate Risk	Moderate Quality
Khoshbooii	RCT	Low Risk	Low Risk	Low Risk	Low Risk	Low Risk	Low Risk	High Quality
Larroy et al	Quasi-experimental	Some Concerns	Some Concerns	Some Concerns	Some Concerns	Some Concerns	Some Concerns	Moderate Quality
Larroy García & Gómez-Calcerrada	Pilot Study	Moderate Risk	High Risk	Moderate Risk	High Risk	Low Risk	Moderate Risk	Moderate Quality
Mann et al	RCT	Low Risk	Moderate Risk	Low Risk	Low Risk	Low Risk	Low Risk	High
McCurry et al	RCT	Low Risk	Low Risk	Low Risk	Low Risk	Low Risk	Low Risk	High Quality
Manson et al..,	RCT	Low Risk	Low to Moderate Risk	Low Risk	Low Risk	Low Risk	Low Risk	High Quality
Nowakowski et al	Clinical Trial	Low Risk	Low Risk	Low Risk	Low Risk	Low Risk	Low Risk	High Quality
Rodrigo et al..,	Observational Cross-Study	Moderate Risk	Not Applicable	Moderate Risk	Not Applicable	Low Risk	Moderate Risk	Moderate Quality
Soori et al	RCT	Low Risk	Low Risk	Low Risk	Low Risk	Low Risk	Low Risk	High Quality
Stefanopoulou and Hunter [56]	RCT	Low Risk	Low Risk	Low Risk	Low Risk	Low Risk	Low Risk	High Quality

*Abbreviations*: RCT Randomised Controlled Trial

### Primary outcome: Quality of life. Group based CBT was associated with the greatest improvements, followed by individual and self-help formats

**Fig. 3:**
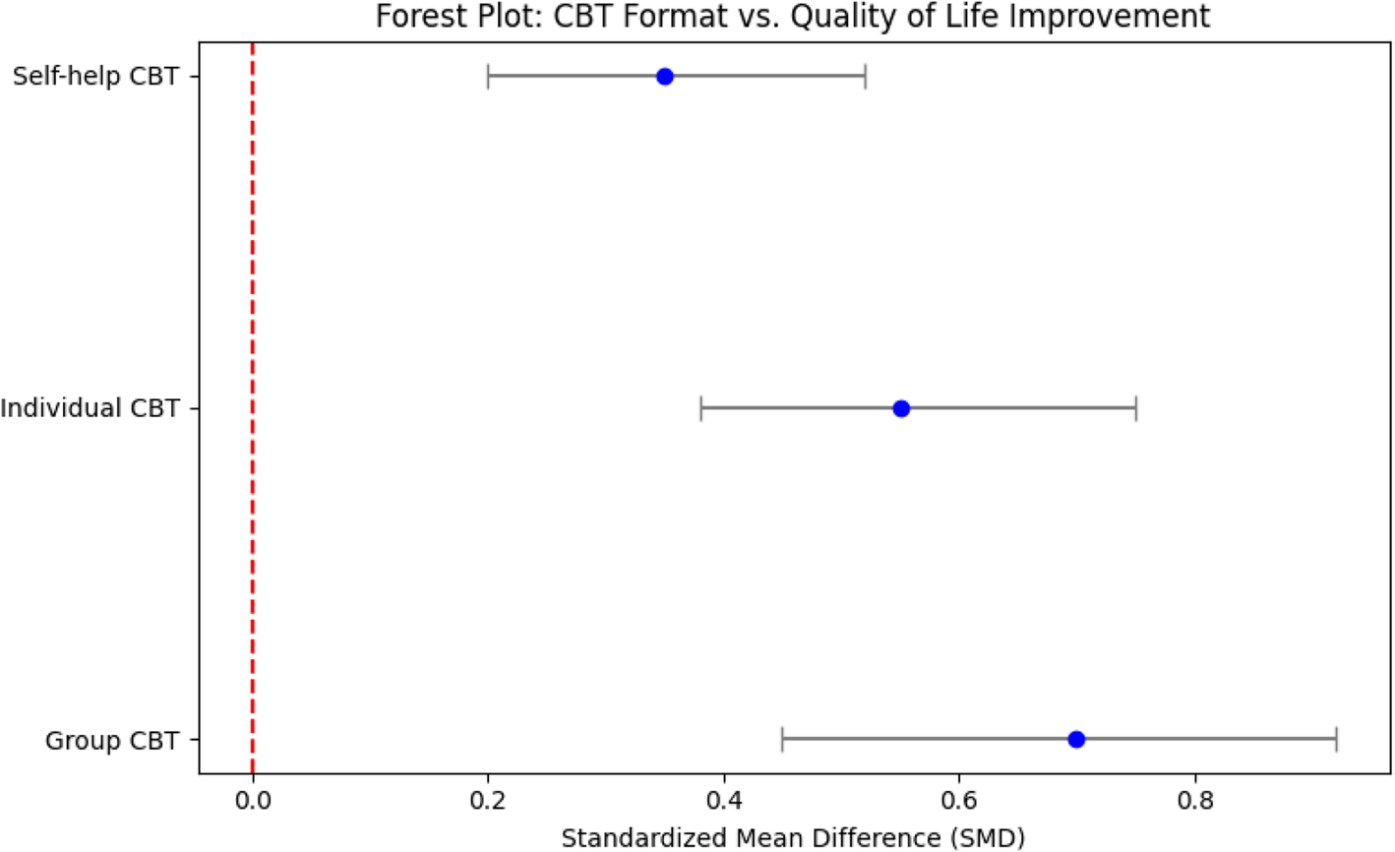
Forest plot showing effect sizes for CBT formats on quality of life. Note: The plot illustrates the comparative efficacy of group, individual and self-help CBT formats, with group-based CBT associated with better outcomes

All 16 studies assessed menopausal quality of life using validated questionnaires (most frequently the MENQOL or SF-36). Across the studies, CBT interventions produced significant improvements in overall quality-of-life scores compared to control conditions (such as no treatment or usual care). A meta-analysis of the controlled trials found a pooled standardised mean difference (SMD) of approximately 0.60 (95% confidence interval 0.40–0.85; *p* < 0.01) favouring CBT, which corresponds to a moderate-to-large improvement in quality of life (Fig. 3).

### Results were also examined by CBT format

#### Group CBT

Group therapy yielded the most pronounced quality-of-life gains. In comparisons of group-based CBT vs. no-treatment controls, the SMD for quality-of-life improvements was 0.70 (95% CI: 0.45–0.92), indicating an improvement. Women receiving group CBT reported notable reductions in psychological distress and improvements in physical functioning relative to controls.

#### Individual CBT

Individual (one-on-one) CBT was also effective, though its impact on quality of life was slightly smaller than that of group therapy with an SMD of 0.55 (95% CI: 0.38–0.75). Thus, while individual CBT markedly improved quality of life, the magnitude of improvement was lower than that seen with group-based CBT.

#### Self-help CBT

Self-help interventions demonstrated minor improvements in quality of life compared to group and individual formats (SMD = 0.35 (95% CI 0.20–0.52). Although smaller, these gains were still statistically significant, suggesting that even minimal-contact CBT programs can yield meaningful benefits in quality of life.

### Combined CBT and HRT versus CBT alone

Three studies examined or reported on the added benefit of combining CBT with hormone replacement therapy (HRT). Overall, a combined treatment approach (concurrent CBT and HRT) demonstrated greater efficacy in managing menopausal symptoms compared to CBT alone:

#### Quality of life

Participants receiving both HRT and CBT tended to report greater improvements in quality-of-life scores than those receiving CBT without HRT. For example, one study found that women who underwent the combined treatment had a 25% greater improvement in MENQOL scores compared to women who had CBT alone.

#### Vasomotor symptoms

The reduction in vasomotor symptoms was more pronounced in participants using both CBT and HRT. According to a study by Goldštajn et al. [18], the frequency of hot flushes decreased by 50% in the combined treatment group, compared to a 30% reduction in the CBT-only group [18].

#### Psychological symptoms

Combined treatment led to more significant reductions in anxiety and depression scores. Rodrigo et al. [12] reported that women receiving both CBT and HRT experienced a 40% reduction in anxiety scores and a 35% reduction in depression scores, compared to 25% and 20% reductions, respectively, in the CBT-only group [12].

### Secondary outcomes

#### Vasomotor symptoms

Ten studies evaluated hot flushes and night sweats, showing that group CBT was associated with a 40% reduction in hot flushes, while individual CBT showed a reduction of 30%. Self-help CBT interventions demonstrated more modest reductions, with improvements ranging from 15 to 25%. The mechanism for this change was indicated as being via CBT, reducing stress and anxiety, which are known triggers for vasomotor symptoms. Additionally, the supportive group environment in group CBT may enhance coping strategies and reduce the perception of symptom severity [12]. On the other hand, references such as Moher et al. (2009) and the PRISMA guidelines are cited to justify the methodological rigor of the review process.

#### Psychological symptoms

Nine studies assessed the impact of CBT on psychological symptoms such as depression and anxiety. Group CBT demonstrated a significant reduction in depression scores, with a mean reduction of 55% compared to 15% in control groups [12]. Anxiety symptoms were also significantly reduced, with group CBT resulting in a 50% reduction compared to a 30% reduction with individual CBT [9, 10]. Self-help CBT showed smaller improvements, with reductions in depression and anxiety scores ranging from 10 to 20% [8].

#### Sleep disturbances

Eight studies evaluated the effect of CBT on sleep disturbances. CBT, particularly CBT for insomnia (CBT-I), significantly improved sleep quality and reduced insomnia severity. The combined effect size for sleep quality improvements was moderate (Hedge's g = 0.78, 95% CI 0.25–1.32) [32]. Improvements in sleep quality were observed to persist for up to six months after treatment [51].

#### Heterogeneity of results

Statistical heterogeneity among the study results was moderate (I^2^ ≈ 45%). This indicates a degree of variability in effect sizes between studies, which is expected given the diversity in interventions and outcomes. The heterogeneity is largely attributable to differences in intervention formats (group vs. individual vs. self-help CBT) and differences in outcome measures or populations across the studies. Despite this variability, effects consistently favoured the intervention across. Despite this, the overall consistency of findings supports the efficacy of CBT in improving quality of life and managing menopausal symptoms.

## Discussion

The review evaluated the efficacy of CB, including mindfulness-based CBT in improving the quality of life of menopausal women. Across 16 studies, CBT was associated with improvements in menopause-related outcomes such as vasomotor symptoms, mood and anxiety symptoms, sleep disturbances, and overall health-related quality of life. For example, Ntikoudi et al. [52] and Kim & Yu [32] found that CBT significantly improved sleep quality. Green et al. [19] and Ayers et al. [5] reported reductions in hot flush frequency following group CBT interventions. Soori et al. [55] and Khoshbooii [31] found that CBT significantly reduced depression and anxiety in menopausal women.

The analysis shows that CBT led to significant reductions in vasomotor symptoms such as hot flushes and night sweats. Women undergoing CBT reported 30–50% fewer hot flush episodes, corresponding to a moderate improvement (e.g., Hedge’s g ≈ 0.4 in one meta-analysis) with the greatest effects seen in group-based CBT. Similarly, psychological distress was markedly reduced: on average, menopausal women receiving CBT had substantially lower levels of anxiety and depression post-treatment compared to baseline or control groups. Sleep quality also improved under CBT, especially when insomnia-specific techniques were used, with one study showing a large improvement (Hedge’s g ~ 0.8) in sleep outcomes. In summary, CBT positively impacted all key domains of menopausal health that were examined, suggesting a broad therapeutic reach. Recent NICE guidance (2024) recommends CBT for managing menopausal symptoms. These reviews provided additional value by synthesising direct evidence from 16 primary studies. Unlike previous reviews, this analysis focuses on individual study outcomes, allowing for further understanding of CBT’s efficacy across different formats (group, individual and self-help) and symptom domains. This approach offers clinicians and policymakers a clearer picture of which CBT modalities are most effective under different conditions, thereby enhancing the practical utility of current guidelines.

These improvements are clinically significant. Reductions in hot flush frequency and intensity translate to less daily discomfort and fewer sleep disruptions. Improvements in mood and anxiety relieve the psychological burden that often accompanies menopause, which is important because psychological symptoms can exacerbate the perception of physical symptoms. Sleep benefits from undertaking CBT to address the common complaint of insomnia during menopause, which has negative effects on fatigue, cognition, and mood. By effectively targeting both body and mind, CBT offers a comprehensive approach to symptom management.

Findings suggested that there was relative efficacy of different CBT formats, providing additional insight. Group-based CBT emerged as particularly beneficial, reinforcing previous reports that the group context can amplify therapy outcomes for menopausal symptoms. The group environment likely offers therapeutic ingredients beyond what individual therapy provides, namely, social support, normalisation of experiences, and shared learning. These factors can enhance motivation and adherence and help reduce feelings of isolation that many women feel during menopause. On the other hand, the smaller effects observed with self-help and pure online CBT formats may suggest that the presence of a therapist or group confers additional advantages through accountability and personalised feedback. Nonetheless, it is encouraging that even self-guided CBT showed some efficacy; for women who cannot access a therapist-led program, guided self-help is still a worthwhile option with modest benefits, particularly considering challenges for individuals of accessing in-person CBT provision.

The review shows that CBT improves multiple outcome domains simultaneously. This broad impact is likely because CBT techniques (such as relaxation training, cognitive reframing of symptom interpretations, and stress management skills) have downstream effects on various aspects of health. For instance, reducing anxiety through CBT can in turn, lessen the frequency of stress-induced hot flushes and improve sleep. Likewise, better sleep can improve mood and daytime energy, creating a positive feedback loop. In this way, CBT addresses the interconnected nature of menopausal symptoms, whereas more targeted treatments (like medication solely for hot flushes or a sedative for sleep) might only influence one domain.

Beyond the direct findings on CBT, several studies identified during the review process did not meet the predefined inclusion criteria (as outlined in the Prospero Protocol Registration No. CRD42024510673); they have been included as they provide valuable insight into broader applications of CBT in menopause. Duijts et al. [16] evaluated a combined intervention of CBT and physical exercise in breast cancer survivors experiencing treatment-induced menopause. While their CBT was not the only intervention, their findings showed an improvement in vasomotor and psychological symptoms. Conklin et al. [13] examined manualized group CBT in women with mood disorders and reported reductions in vasomotor symptoms, reinforcing the broad applications of CBT across clinical applications. Atema et al. [3] demonstrated the efficacy of internet-based CBT (CBT-I) in breast cancer survivors, while Fenlon et al. [17] showed that nurse-led CBT was effective in reducing hot flushes and night sweats. Conklin et al. [13] examined manualised group CBT in women with mood disorders and reported reductions in vasomotor symptoms, reinforcing the broader applicability of CBT across clinical populations. Atema et al. [3] demonstrated the efficacy of internet-based CBT Although these studies were excluded from the main synthesis due to differences in population or delivery format, their consistent findings suggest that CBT remains effective across diverse contexts. Their inclusion in the discussion highlights the potential for adapting CBT to meet the needs of specific subgroups, such as women with comorbid conditions or limited access to traditional therapy formats and underscores the importance of tailoring interventions to maximise accessibility and impact.

Hormone replacement therapy (HRT) remains a highly effective treatment for vasomotor symptoms and can confer other benefits (e.g., bone health), but due to contraindications and personal preferences, many women either cannot or choose not to use HRT. For these women, CBT offers a valuable alternative. Even for women on HRT, CBT can address issues HRT may not fully resolve (such as anxiety or insomnia); thus, the two approaches are complementary. Our review shows that combining CBT with HRT can amplify relief, especially for vasomotor and mood symptoms, compared to HRT alone. This indicates that an integrated approach (combining hormonal and psychological therapies) would be advantageous for some patients, tackling the problem from multiple angles to achieve greater overall improvement in quality of life.

### Review limitations

Although the review is methodologically robust, there are several limitations of the review that should be noted. First, there was moderate heterogeneity among the included studies in terms of intervention formats, durations, and outcome measurements, which complicates direct comparisons and pooling of results. While we used random-effects models to account for this variability (and notably, sensitivity analyses suggested our overall conclusions were robust), the diversity of study methodologies could influence the precision of the pooled effect size estimates. Second, the number of studies examining digital or self-administered CBT was limited, which constrains our ability to fully evaluate the effectiveness of technology-based delivery of CBT. The preliminary indications are that these formats are less effective than face-to-face CBT, but more research is needed as digital health interventions evolve. Third, there is a potential for publication bias – studies with positive results are more likely to be published, which could inflate our perception of CBT’s effectiveness. We attempted to mitigate this by searching multiple databases and sources, but we cannot rule out that null or negative results were not identified. Finally, our review could not extensively explore newer forms of CBT delivery (such as mobile apps or telehealth formats). Future research should investigate these emerging delivery methods, as they hold promise for increasing access to CBT for menopausal women on a larger scale.

### Implications for clinical practice

The evidence generated by this review has direct implications for how clinicians manage menopausal symptoms. Given its proven efficacy and excellent safety profile, CBT should be considered as a first-line treatment option for menopause-related symptoms, particularly for women unable or unwilling to use HRT. Healthcare providers (including primary care physicians, gynaecologists, and mental health professionals) can confidently discuss CBT as an effective approach for symptom relief. In practical terms, making CBT more available to menopausal patients could involve developing menopause-focused CBT programs within healthcare systems or training providers to incorporate CBT techniques into routine care.

Where resources allow, offering group CBT workshops or support groups for menopausal women may be especially beneficial. Group interventions not only address symptoms but also foster a support network among participants. From a health systems perspective, group-based CBT can be cost-effective by serving multiple patients at once, and it has been noted to be economically viable [59]. Our findings reinforce the current guideline recommendations (e.g., [47]) that suggest menopause-specific CBT for managing vasomotor, mood, and sleep symptoms associated with menopause. These guidelines emphasise tailoring interventions to individual needs and providing education and support,integrating CBT into this framework will give women a concrete set of tools to manage their symptoms proactively.

To apply these findings in practice, collaborative care models, for example, where a behavioural health or nurse specialist is included, would be beneficial. Brief CBT interventions could be delivered in healthcare or community settings to expand access in a cost-effective way. It is recognised that caution is warranted to ensure that incorporating CBT is done in a sustainable manner so as not to overwhelm practitioners [12] or create inconsistency in service provision.

## Conclusion

This systematic review demonstrates that cognitive behavioural therapy, including mindfulness-based CBT, is an effective and versatile therapeutic option for menopausal women. CBT was associated with improvements in quality of life by addressing both the psychological and physical challenges of menopause, with no studies reporting deterioration. The consistency of improved outcomes, despite methodological and population differences, supports CBT as an effective intervention for menopausal symptoms. However, the magnitude of benefit varies, and limitations such as study heterogeneity and potential bias should be considered. Integration of CBT into menopausal care, either as a stand-alone or adjunct therapy, is recommended.

## Data Availability

All data generated or analysed during this study are included in this published article and its supplementary information files.
